# Contribution and Interactions of Hydroxycinnamic Acids Found in Bran and Wholegrain Sorghum (*Sorghum bicolor* L. Moench): Effects on the Antioxidant Capacity and Inhibition of Human Erythrocyte Hemolysis

**DOI:** 10.1155/2017/8219023

**Published:** 2017-10-12

**Authors:** Norma Julieta Salazar-López, Gustavo A. González-Aguilar, Guadalupe Loarca-Piña, Francisco J. Cinco-Moroyoqui, Ofelia Rouzaud-Sández, J. Abraham Domínguez-Avila, Maribel Robles-Sánchez

**Affiliations:** ^1^Departamento de Investigación y Posgrado en Alimentos, Universidad de Sonora, C.P. 83000 Hermosillo, SON, Mexico; ^2^Centro de Investigación en Alimentación y Desarrollo A.C., C.P. 83304 Hermosillo, SON, Mexico; ^3^Departamento de Investigación y Posgrado en Alimentos, Facultad de Química, Universidad Autónoma de Querétaro, C.P. 76010 Santiago de Querétaro, QRO, Mexico

## Abstract

An imbalance between free radicals and antioxidants is known as oxidative stress, and it promotes cellular aging and the development of chronic noncommunicable diseases. The bioactive compounds present in food play an important role in preventing oxidative stress. The aim of this study was to determine the contributions and interactions of the hydroxycinnamic acids found in the bran and whole grain of sorghum and to evaluate their effects on the antioxidant capacity and inhibition of the hemolysis of human erythrocytes. Results showed that the caffeic acid, *p*-coumaric acid, and ferulic acid found in sorghum contributed to the scavenging of DPPH and ABTS radicals in various proportions. Ferulic acid, which was present in bound form in the bran and wholegrain sorghum, significantly inhibited the AAPH radical-induced oxidation of the erythrocyte membranes by 78.0 and 4.3%, respectively. Combinations of two, three, or four hydroxycinnamic acids may interact in an antagonistic or synergistic manner, thereby altering each other's bioactivities. The various interactions between the different sorghum bioactives can have a significant impact on their potential bioactivities. These results can be useful in the design of functional foods that aim to deliver bioactives to mitigate cellular aging or noncommunicable diseases.

## 1. Introduction

An imbalance between free radicals and antioxidants in the cell is known as oxidative stress, which favors cellular aging and the development of some chronic noncommunicable diseases including diabetes, cardiovascular disease, and some types of cancer, among others [1–3]. Dietary choices can mitigate oxidative stress by delivering bioactive compounds with high antioxidant capacity, thereby inhibiting the development and progression of cellular damage. Some cereals are sources of phenolic compounds with a high antioxidant capacity that can efficiently transfer electrons and/or hydrogen atoms. In addition, these cereals maintain health by modulating numerous cellular processes [4]. Sorghum (*Sorghum bicolor* L. Moench) is a source of phenolic compounds with low molecular weights and simple structures, specifically hydroxycinnamic acids [5, 6]. Among these compounds, ferulic acid is present at the highest concentration, and it is found both in its free form and covalently bound to the arabinoxylans that are present in the cell walls of the endosperm, aleurone layer, and pericarp [6–9]. In humans, bound ferulic acid is poorly bioaccessible and bioavailable in the small intestine because the indigestible matter transports it to the large intestine, where it can be metabolized by the resident microbiota. The arabinoxylan matrix can be modified before consumption by acid or basic hydrolysis, fermentation, extrusion, or other processes. These processes yield free ferulic acid or feruloylated oligosaccharides in which the bioaccessibility and bioavailability of ferulic acid is improved [10]. These methods can be used to improve the bioactivity of sorghum and can be used in the development of functional foods.

The bioactivity can also be modified by physicochemical interactions among the various hydroxycinnamic acids in the cereal, with other molecules in the food matrix or with molecules in coconsumed foods. These interactions can produce synergistic, antagonistic, or additive effects, thereby increasing or decreasing the net antioxidant activity.

The aim of this study was to determine the individual contributions of the main hydroxycinnamic acids found in the sorghum bran (SB) and wholegrain sorghum (WG) to the total antioxidant capacity as well as interactions among these compounds and their effects on the inhibition of human erythrocyte hemolysis. As far as we know, no previous studies that included evaluations of the antioxidant capacity and inhibition of hemolysis as indicators of the bioactivity have been performed on sorghum.

## 2. Materials and Methods

### 2.1. Materials and Chemicals

Ferulic acid, caffeic acid, sinapic acid, *p*-coumaric acid, Folin-Ciocalteu reagent, 2,2-diphenyl-1-picrylhydrazyl (DPPH), (±)-6-hydroxy-2,5,7,8-tetramethylchromane-2-carboxylic acid (Trolox), 2,2′-azino-bis(3-ethylbenzothiazoline-6-sulfonic acid) diammonium salt (ABTS), ammonium persulfate, and 2,2′-azobis(2-amidinopropane) dihydrochloride (AAPH) were purchased from Sigma-Aldrich, St. Louis, MO, USA. Hydrochloric acid, ethanol, sodium hydroxide, and methanol were purchased from J.T. Baker, Mexico State, Mexico.

### 2.2. Preparation of Sorghum Samples

An unpigmented sorghum variety (UDG110) was kindly donated by “Fundación Produce,” Mexico. The grains were milled (Pulvex 200 mill) and sieved (0.4 mm). The SB was obtained by sorghum decortication using abrasive discs for 6 min, and the WG was ground to the same particle size; both were stored at −20°C until analysis.

### 2.3. Extraction of Free and Bound Phenolics

The extraction of the free phenolics (FP) was conducted according to Salazar-Lopez et al. [11]. Briefly, 1 g of either WG or SB was accurately weighed in a screw-capped tube, and the FP were then extracted with 15 mL of 80% methanol, after which each sample was sonicated separately for 1 h and centrifuged at 1500*g* for 15 min in a benchtop centrifuge to precipitate the solids. This procedure was performed twice (30 min total); the three supernatants were mixed and filtered using Whatman number 1 filter paper. The filtrates were evaporated to dryness under vacuum at 35°C and resuspended in 50% methanol-water to a final concentration of 200 mg/mL. The obtained samples were named FPW or FPB for free phenolics from wholegrain or free phenolics from bran, respectively.

For the extraction of bound phenolics (BP), an alkaline hydrolysis was initially performed on the dry residues left after the FP were extracted. Briefly, 100 mg of the dry residue was hydrolyzed for 3 h at room temperature with 2 N or 4 N NaOH for the WG or SB, respectively. The pH was adjusted (1.5–2) using 6 N HCl. Liquid-liquid extractions were performed using ethyl acetate; the organic layer was recovered and evaporated under vacuum at 35°C, and the residue at the bottom of the flask was resuspended in 5 mL of 50% methanol to a final concentration of 20 mg/mL. These samples were named BPW or BPB for bound phenolics from wholegrain or bound phenolics from bran, respectively [12].

### 2.4. Quantification of Total Phenolics

The total phenolics were quantified using the Folin-Ciocalteu reagent as described by Singleton and Rossi [13], with modifications to permit reading the results in a microplate reader (FLUOstar Omega, BMG Labtech Inc., Ortenberg, Germany). Briefly, 30 *μ*L of each extract was mixed with 150 *μ*L of the Folin-Ciocalteu reagent (previously diluted 1 : 10 with deionized water) in a microplate well, after which 120 *μ*L of sodium carbonate solution (0.075 g/mL) was added and thoroughly mixed. The samples were allowed to react in the dark for 30 min, and the absorbance was read at 765 nm [14]. A calibration curve was performed in parallel with the samples using gallic acid as a standard for use in determining the concentration of total phenolics. The results are expressed as *μ*g of gallic acid equivalents (GAE)/g of the sample.

### 2.5. Chromatographic Identification and Quantification of Hydroxycinnamic Acids

The hydroxycinnamic acids were identified and quantified using an Agilent Technologies UHPLC system with a diode array detector (UHPLC-DAD). The separation was carried out using a Zorbax Eclipse Plus C_18_ rapid resolution column (50 mm × 2.1 mm i.d. 1.8 *μ*m particle size). A binary phase solvent system was used: solvent A was 0.1% acetic acid dissolved in water, and solvent B was 0.1% acetic acid dissolved in methanol. The solvent gradient was as follows: 0–11 min, 9 to 14% B and 11–15 min, 15% B. The column temperature was set at 30°C, the flow rate was 0.7 mL/min, and the detector was set at 280 nm. The results were expressed as *μ*g/g of dry weight (DW) as determined using methanolic solutions (1.5–50 *μ*g/mL) of caffeic acid, *p*-coumaric acid, ferulic acid, and sinapic acid as standards [11].

### 2.6. Trolox Equivalent Antioxidant Capacity (TEAC) Assay

The Trolox equivalent antioxidant capacity (TEAC) assay is based on the ability of antioxidant molecules to scavenge the ABTS cation radical, which produces a change in its color that can be spectrophotometrically quantified [15]. A stable stock solution of ABTS was prepared by mixing 5 mL of an aqueous solution of ABTS (7 mM) with 0.088 mL of sodium persulfate (148 mM) and incubating it in the dark at room temperature for 16–18 h. The ABTS working solution was prepared immediately before use by diluting the stock solution in ethanol (∼1 : 88, *v*/*v*), and its absorbance was adjusted to 0.7 ± 0.02 at 734 nm. Then, 280 *μ*L of the ABTS working solution was combined with 10 *μ*L of the sample of interest in a microplate well. The changes in absorbance (734 nm) were recorded as ABTS radical-scavenging activity. A standard curve was prepared using Trolox as a standard, which was used to convert the changes in absorbance of the samples to *μ*mol of Trolox equivalents (TE)/g of the sample.

### 2.7. DPPH Assay

The DPPH radical-scavenging assay was performed as described by Robles-Sánchez et al. [16]. The assay is based on the scavenging of the DPPH radical, which changes its coloration from dark purple to yellow. The changes in absorbance in the 515–520 nm range correlate with the antioxidant capacity of the molecules being analyzed. For this assay, 20 *μ*L of each sample was pipetted into a microplate well, and 280 *μ*L of a methanolic DPPH solution (0.025 mg/mL) was added. The mixtures were incubated with constant shaking for 30 min at room temperature in the dark, and the changes in absorbance were subsequently monitored at 515 nm. A standard curve of Trolox was prepared, and the changes in absorbance of the samples were converted to *μ*mol TE/g of the sample.

### 2.8. Inhibition of Human Erythrocyte Hemolysis

To determine the ex vivo effects of the extracts, they were initially evaporated to dryness under a nitrogen stream. The dry samples were resuspended in phosphate-buffered saline (PBS 150 mM, pH 7.4) to a final concentration of 200 mg/mL.

The antioxidant activity of the extracts was measured as the percentage of erythrocyte hemolysis [17]. Briefly, 5 mL of peripheral blood from healthy adult volunteers was collected in EDTA tubes. The erythrocytes were separated from the plasma and the buffy coat, washed three times with 10 mL of PBS, and centrifuged at 1500*g* for 5 min. The erythrocytes were obtained in the final wash after a centrifugation at 1500*g* for 10 min.

To perform the assay, 100 *μ*L aliquots of a 10% erythrocyte suspension (0.6 ± 0.1 × 10^6^ cells/mL) were incubated with 25 *μ*L of the extracts for 20 min at 37°C. After that, 50 *μ*L of a 300 mM AAPH solution was added, and the mixture was incubated at 37°C for 5 h with gentle shaking. The samples were then diluted with 2.1 mL of PBS and centrifuged for 10 min at 1500*g*. The supernatants were recovered and their absorbances were read at 540 nm (*A*_Ext_). Positive (*A*_AAPH_) and negative hemolysis controls were carried out as follows: the positive control included a suspension of erythrocytes and the AAPH solution, whereas the negative control contained only the erythrocyte suspension. Furthermore, an extract control was included, in which PBS was added in place of the AAPH solution.

The percentage of hemolysis was calculated according to the following equation:

(1) $$\begin{array}{c} \%\;of\;hemolysis=\left[\frac{A_{Ext}}{A_{AAPH}}\right]\times 100. \end{array}$$

The assays were carried out in triplicate, and the results were expressed as % of hemolysis.

### 2.9. Contribution of Hydroxycinnamic Acids to Total Antioxidant Capacity

The contribution of the individual hydroxycinnamic acids found in the FPW, FPB, BPW, and BPB extracts was evaluated through the TEAC, DPPH, and hemolysis inhibition assays. A range of concentrations (0.001–1 mg/mL) of each standard was analyzed, the equations for the linear regressions were obtained, and the IC_50_ (*μ*mol/mL) was determined for each hydroxycinnamic acid. The contribution (%) of each compound to the total antioxidant capacity of the FPW, FPB, BPW, and BPB samples was determined by replacing the results of antioxidant capacity (DPPH and TEAC) and hemolysis inhibition in the linear regression equations.

### 2.10. Interactions between Hydroxycinnamic Acids

To identify additive, synergistic, or antagonistic interactions among the hydroxycinnamic acids, the method suggested by Palafox-Carlos et al. [18] was used with slight modifications. Briefly, commercial standards of caffeic (A), *p*-coumaric acid (B), ferulic acid (C), and sinapic acid (D) were prepared (stock solutions of 0.25 mM each in PBS). The antioxidant capacity of each combination of two, three, and four compounds was determined using the TEAC and DPPH methods as previously described. To evaluate the type of interaction between the compounds, two values of antioxidant capacity were used: (a) the theoretical value, defined as the sum of the antioxidant capacity values of the individual standard in each combination; (b) the real value, which corresponded to the experimentally quantified antioxidant capacity of each combination. Thereafter, the theoretical and real values were compared to determine whether significant additive, synergistic, or antagonistic interactions had occurred.

### 2.11. Statistical Analysis

To verify the statistical significance of all obtained results, the means ± SD were calculated using the statistical software JMP 5.0.1 (Statistical Discovery™, SAS). The antioxidant capacities, percentages of hemolysis inhibition, and concentrations of compounds were tested using an ANOVA and a Tukey test. *p* values less than 0.05 were considered statistically significant.

## 3. Results and Discussion

### 3.1. Concentration of Phenolics and Hydroxycinnamic Acids

Table 1 summarizes the total phenolic concentrations (TPC) and hydroxycinnamic acid concentrations (HCA) of the free and bound phenolics from the wholegrain extracts (FPW; BPW) and the free and bound phenolics from the bran extracts (FPB; BPB). The TPC values of the FPB (2022.2 ± 31.4 *μ*g GAE/g) and BPB (7425.0 ± 318.7 *μ*g GAE/g) extracts were significantly higher than those of the FPW (784.3 ± 29.2 *μ*g GAE/g) and BPW (2107.9 ± 40.3 *μ*g GAE/g) extracts. These results showed that 72.88% and 79.28% of the total phenolics from the WG and SB were recovered after alkaline hydrolysis. Similar results have been reported for other cereals including corn, oat, wheat, and red sorghum, for which the percentages of bound phenolics are 85, 75, 75, and 85%, respectively [19]. The percentages of BP are significantly less in other food matrices such as apples (6.5%) and oranges (24.3%), which demonstrates that the structural arrangement of the phenolic compounds is more complex in cereals than in fruits [19].

The identification and quantification of the hydroxycinnamic acids in the WG and SB samples were obtained by comparing their absorption spectra and retention times (1.96, 3.56, 5.70, and 8.04 min for caffeic acid, *p*-coumaric acid, ferulic acid, and sinapic acid, resp.) to those of commercial standards. We determined that in the bound fraction of both WG and SB, ferulic acid was found at a significantly higher concentration than the other hydroxycinnamic acids that were quantified (*p* < 0.05) (Table 1).

Our results for the HCA content in WG and SB are in agreement with those of Chiremba et al. [6], who reported that the highest proportion of phenolic compounds in cereals are bound and that ferulic acid is the major hydroxycinnamic acid in sorghum [6, 7]. The concentrations of caffeic acid and sinapic acid were minimal in the FP fractions of both WG and SB; neither compound was detected in the bound fraction. These results are similar to those reported by Krygier and Hogge [20], who obtained losses of 66.7 and 36.5% of caffeic acid and trans-sinapic acid after incubation in alkaline conditions (2 N NaOH for 4 h at room temperature), whereas ferulic acid and *p*-coumaric acid were much less affected (losses of 4.8 and 2.7%, resp.) [20]. A more recent study by Verma et al. [21] showed that caffeic acid and syringic acid were also affected during alkaline extraction. This could be explained because alkaline conditions induce the oxidation and dimerization of these hydroxycinnamic acids, thereby decreasing their concentration [6].

### 3.2. Antioxidant Capacity

**Figure 1:**
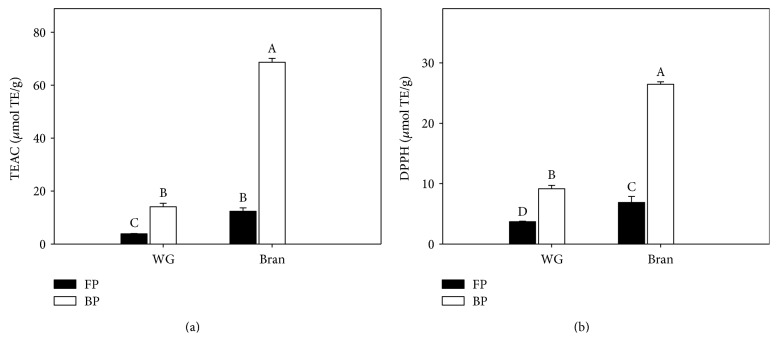
Antioxidant capacity of the free phenolic (FP) and bound phenolic (BP) extracts of wholegrain sorghum (WG) and sorghum bran: (a) TEAC assay and (b) DPPH assay. Each bar represents the mean of three replicates ± standard deviation. Different superscript letters in the bars represent significant differences (*p* < 0.05) between free and bound fractions from WG and SB.

Figure 1 depicts the antioxidant capacity of the free phenolic and bound phenolic extracts of wholegrain (FPW, BPW) and sorghum bran (FPB, BPB), respectively, as determined by the TEAC (1A) and DPPH (1B) assays. Significant differences were observed for the scavenging capacity of the sorghum extracts towards the DPPH and ABTS∗ radicals. The FPB and BPB samples showed a significantly higher scavenging capacity for the ABTS∗ radical (12.36 ± 1.29 and 68.69 ± 1.42 *μ*mol TE/g, resp.) than the FPW and BPW samples (3.84 ± 0.13 and 14.08 ± 1.30 *μ*mol TE/g, resp.). A similar behavior was determined in the DPPH assay, in which the BP from both extracts (BPW and BPB) also demonstrated higher DPPH radical-scavenging activity.

The results for the antioxidant capacity of WG and SB are consistent with those obtained for the TPC (Table 1). It can be argued that most of the antioxidant capacity of the SB is attributable to the hydroxycinnamic acids that are bound to arabinoxylans. Several studies have shown low bioaccessibility, particularly of ferulic acid, after high bran consumption [22, 23]. This low ferulic acid bioaccessibility in products prepared with the bran of several cereals also decreases its bioavailability, up to 3% in humans and 2.5–5.5% in rats. Furthermore, its bioavailability in corn bran is much lower, approximately 0.4–0.5%, which could be explained on the basis that the complexity of the corn matrix that contains the ferulic acid is greater than that of the matrices of wheat and barley. In corn and sorghum, ferulic acid is bound to a more substituted chain of arabinoxylans that limits the access and activity of digestive esterases [9, 23, 24].

### 3.3. Inhibition of Induced Hemolysis of Human Erythrocytes

**Figure 2:**
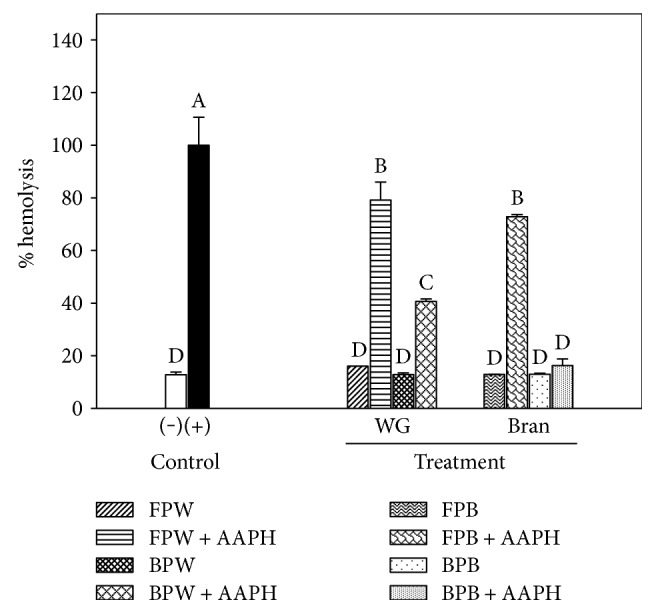
Hemolysis (%) of the free phenolic (FP) and bound phenolic (BP) extracts of wholegrain sorghum (WG) and sorghum bran. Each bar represents the mean of three replicates ± standard deviation. Different superscript letters in the bars represent significant differences (*p* < 0.05) between free and bound fractions from WG and SB.

The addition of AAPH, a peroxyl radical (ROO^−·^) initiator, causes oxidation of lipids and proteins in the erythrocyte membrane, which promotes its breakdown and causes hemolysis [25].  Figure 2() shows the effects of the various treatments on the hemolysis of human erythrocytes. The BPW and BPB extracts demonstrated a greater inhibition of hemolysis (59.3 and 83.7%, resp.) than the FPW and FPB extracts (20.0 and 24.6%), which showed that the BP extracts provided better protection against erythrocyte membrane oxidation induced by AAPH. Shelembe et al. [26] showed that SB extracts that were obtained under aqueous-acidic conditions inhibited up 50% the hemolysis of human erythrocytes. The higher protective effect of BPW and BPB could be due to their significantly higher TPC levels than those of the FPW and FPB. The protective effect may be explained by the stabilization of the peroxyl radical by the phenolic compounds by the transfer of hydrogen atoms from the hydroxyl groups of the phenolic compounds or by electron transfer from the phenoxide anion. However, at physiological pH (7.4), it is more likely that the primary mechanism by which the hydroxycinnamic acids stabilize the peroxyl radical involves electron transfer from the phenoxide anion [4]. The results suggest that the phenolic compounds in the SB extracts have the potential to reduce oxidative stress on the cell membrane.

### 3.4. Contribution of the Hydroxycinnamic Acids to the Total Antioxidant Capacity and Inhibition of Human Erythrocyte Hemolysis

The antioxidant capacities and inhibition of human erythrocyte hemolysis by pure hydroxycinnamic acids were evaluated.  Table 2 shows the IC_50_ results for each compound in the DPPH, TEAC, and hemolysis inhibition assays. It was observed that at equimolar concentrations, caffeic acid had the greatest capacity to scavenge the DPPH radical, followed by sinapic acid, ferulic acid, and *p*-coumaric acid. A different behavior was observed in the ABTS assay, in which the following order for the radical-scavenging effects were observed: *p*-coumaric acid > ferulic acid > sinapic acid > caffeic acid. Sinapic acid exerted the most hemolysis inhibition followed by caffeic acid, ferulic acid, and *p*-coumaric acid. The differences among the results for the various antioxidant capacity assays could be explained by the nature of each radical and the different kinetics of electron or hydrogen atom transfer of each compound. It has been reported that at a physiological pH, the rate of the reaction between caffeic acid and the peroxyl radical is higher than that for ferulic acid, followed by that for *p*-coumaric acid. This is explained by the presence of a catechol group in caffeic acid and a methoxy group in ferulic acid. These groups allow greater reactivity with the peroxyl radical than the reactivity of *p*-coumaric acid, which has no other groups adjacent to the phenoxide [4]. The results showed different behaviors of the various hydroxycinnamic acids in each assay; whereas caffeic acid had the most DPPH-scavenging effect, *p*-coumaric acid was the most effective ABTS scavenger and sinapic acid exerted the greatest inhibition of hemolysis.

**Figure 3:**
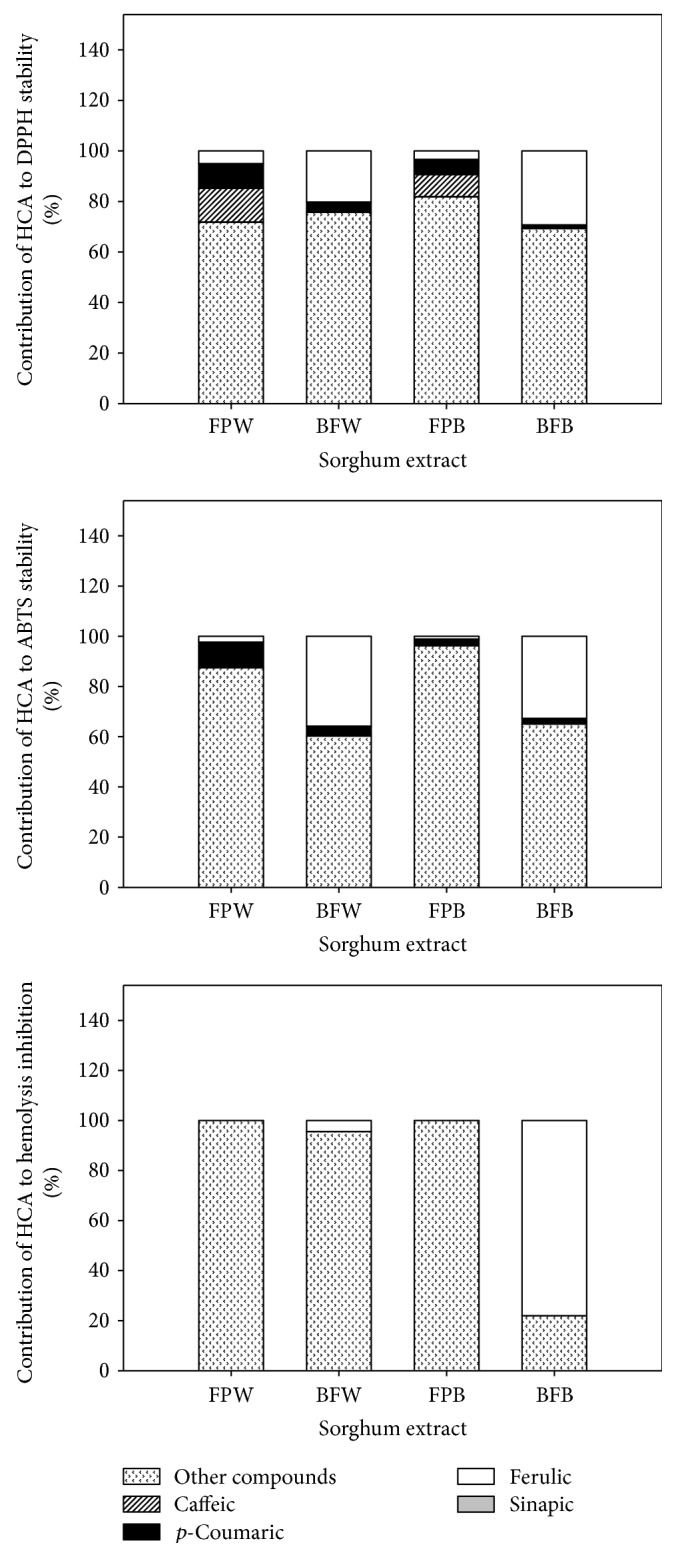
Contribution of hydroxycinnamic acids from sorghum to the total antioxidant capacity and inhibition of human erythrocyte hemolysis. The inhibition of hemolysis was analyzed for equal concentrations of the sorghum extracts (200 *μ*g sorghum extract/mL). Each value represents the mean of three replicates ± standard deviation.

Figure 3 summarizes the contribution of each hydroxycinnamic acid to the total antioxidant capacities and hemolysis inhibition. We observed significant differences in the contributions of these compounds to the three assays evaluated (DPPH, TEAC, and hemolysis inhibition).

The contributions to the antioxidant capacity (DPPH) due to hydroxycinnamic acids present in the BP fraction were 30.72% and 24.30% for BPB and BPW, respectively. In both cases, ferulic acid and *p*-coumaric acid were the major contributors (29.0 versus 1.4% for SB and 20.3% versus 4.0% for WG). A similar behavior was found in the TEAC assay, in which the contribution percentages for the hydroxycinnamic acids were 34.9% and 39.7% for the BPB and BPW extracts, respectively. Once again, ferulic acid and *p*-coumaric acid were the main contributors to the total antioxidant capacity of the BP extracts.

In the hemolysis inhibition assay, only ferulic acid contributed to the effect (78.0% and 4.3% for BPB and BPW, resp.), because ferulic acid was the only compound present at a concentration sufficient to scavenge the AAPH radical used to induce hemolysis. Although our results showed that the monomeric forms of each hydroxycinnamic acid contributed to the total antioxidant capacity in each extract, it is important to consider that other forms present, including ferulate residues, 3-deoxyanthocyanins, and tannins, could also contribute to the antioxidant capacity of sorghum [5, 27].

### 3.5. Interactions between Hydroxycinnamic Acids

We analyzed the antioxidant capacity of the individual hydroxycinnamic acids at equimolar concentration (0.25 mM) and their interactions. The results are summarized in Table 3. The combinations of two, three, or four hydroxycinnamic acids produced changes in the total antioxidant capacity. The *p*-coumaric acid and ferulic acid combination showed a synergistic interaction in the TEAC assay; the combination of caffeic acid and sinapic acid also showed a synergistic interaction in the DPPH assay. The interaction of caffeic acid with *p*-coumaric acid showed antagonist effects on both assays. The various interactions between caffeic acid, *p*-coumaric acid, ferulic acid, and sinapic acid showed that the presence of two or more compounds in the food matrix could increase their bioactivities. These results allow us to understand the different types of interactions that can occur among the four acids studied here, and this information could be further used in the development of functional foods to avoid undesired interactions and promote desired ones.

## 4. Conclusion

Our results showed that the potential bioactivities of the hydroxycinnamic acids found in wholegrain sorghum and sorghum bran can mainly be attributed to those present in the bound form. The antioxidant capacity assays showed that caffeic acid, *p*-coumaric acid, and ferulic acid contributed to DPPH and ABTS radical scavenging. However, in the ex vivo assay, the results showed that ferulic acid was the main contributor. In addition, the presence of different hydroxycinnamic acids might have favored their interactions, which could be synergistic, antagonistic, or additive. The hydroxycinnamic acids from sorghum play an important role in its potential biological activities. This information could be further used in the development of functional foods to avoid undesired interactions and promote desired ones.

## Figures and Tables

**Table 1:** Total phenolic content and hydroxycinnamic acid content of the free and bound fractions from WG and SB.

Sample	TPC	Total HCA	Caffeic	*p-*Coumaric	Ferulic	Sinapic
FPW	784.3 ± 29.2^c^	42.6 ± 1.10^c^	10.2 ± 1.0^b^	18.1 ± 1.8^c^	12.2 ± 0.2^d^	2.1 ± 0.0^b^
BPW	2107.9 ± 40.3^b^	718.3 ± 12.49^b^	ND	38.7 ± 0.2^b^	679.6 ± 12.3^b^	ND
FPB	2022.2 ± 31.4^b^	46.4 ± 0.90^c^	14.8 ± 0.5^a^	8.6 ± 0.4^d^	19.6 ± 0.4^c^	3.4 ± 0.1^a^
BPB	7425.0 ± 318.7^a^	3205.0 ± 90.69^a^	ND	169.0 ± 3.5^a^	3036.0 ± 87.5^a^	ND

FPW: free phenolics from wholegrain sorghum; FPB: free phenolics from sorghum bran; BPW: bound phenolics from wholegrain sorghum; BFB: bound phenolics from sorghum bran; TPC: total phenolic content (*μ*g GAE/g); Total HCA: total hydroxycinnamic acids content (*μ*g/g); ND: not detected. Each value is the mean of three replicates ± the standard deviation (SD). Different superscript letters in the same column represent significant differences (*p* < 0.05) between free and bound fractions from WG and SB.

**Table 2:** Linear regression and IC_50_ of hydroxycinnamic acids in the antioxidant capacity assays and the inhibition of erythrocyte hemolysis.

HCA	DPPH			ABTS			% Hemolysis inhibition		
	Equation	*R* ^2^	IC_50_	Equation	*R* ^2^	IC_50_	Equation	*R* ^2^	IC_50_
Caffeic	*y* = 0.9853*x* + 13.642	0.881	0.20	*y* = 0.7799*x* − 3.0388	0.987	0.38	*y* = 0.3242*x* − 93.957	0.942	2.46
*p*-Coumaric	*y* = 0.0182*x* + 12.404	0.892	>7.5	*y* = 0.8509*x* + 11.521	0.938	0.28	*y* = 0.1114*x* − 33.291	0.978	4.55
Ferulic	*y* = 0.3624*x* + 5.4449	0.934	0.63	*y* = 0.8674*x* + 1.7052	0.986	0.29	*y* = 0.1331*x* − 15.528	0.929	2.54
Sinapic	*y* = 0.5561*x* + 2.9464	0.987	0.38	*y* = 0.6816*x* − 1.2081	0.975	0.34	*y* = 0.2012*x* + 20.052	0.976	0.66

HCA: hydroxycinnamic acid; IC_50_: half of the maximum inhibitory concentration, expressed as *μ*mol of HCA/mL.

**Table 3:** Antioxidant capacity of the hydroxycinnamic acids found in sorghum and their interactions.

	TEAC			DPPH		
	Real	Theoretical (sum)	Type of interaction	Real	Theoretical (sum)	Type of interaction
*Individual*						
A (caffeic)	0.26 ± 0.02^b^			0.38 ± 0.03^a^		
B (*p*-coumaric)	0.45 ± 0.02^a^			0.02 ± 0.00^d^		
C (ferulic)	0.46 ± 0.04^a^			0.22 ± 0.02^c^		
D (sinapic)	0.32 ± 0.03^b^			0.29 ± 0.01^b^		
*Interactions*						
AB	0.58 ± 0.02^b^	0.71 ± 0.02^a^	Antagonistic	0.34 ± 0.03^b^	0.40 ± 0.00^a^	Antagonistic
AC	0.62 ± 0.01^b^	0.72 ± 0.04^a^	Antagonistic	0.62 ± 0.04^a^	0.60 ± 0.02^a^	Additive
AD	0.43 ± 0.02^b^	0.59 ± 0.03^a^	Antagonistic	0.72 ± 0.02^a^	0.67 ± 0.01^b^	Synergistic
BC	1.06 ± 0.06^a^	0.90 ± 0.04^b^	Synergistic	0.22 ± 0.02^a^	0.24 ± 0.02^a^	Additive
BD	0.76 ± 0.05^a^	0.77 ± 0.03^a^	Additive	0.32 ± 0.02^a^	0.31 ± 0.01^a^	Additive
CD	0.80 ± 0.05^a^	0.78 ± 0.03^a^	Additive	0.53 ± 0.05^a^	0.51 ± 0.01^a^	Additive
ABC	0.99 ± 0.09^b^	1.16 ± 0.03^a^	Antagonistic	0.55 ± 0.04^b^	0.62 ± 0.02^a^	Antagonistic
ACD	1.04 ± 0.03^a^	1.04 ± 0.06^a^	Additive	0.86 ± 0.03^a^	0.89 ± 0.03^a^	Additive
BCD	1.13 ± 0.02^a^	1.23 ± 0.06^a^	Additive	0.46 ± 0.01^b^	0.53 ± 0.03^a^	Antagonistic
BDA	0.99 ± 0.03^a^	1.03 ± 0.02^a^	Additive	0.62 ± 0.03^a^	0.69 ± 0.04^a^	Additive
ABCD	1.32 ± 0.06^b^	1.49 ± 0.04^a^	Antagonistic	0.88 ± 0.03^a^	0.91 ± 0.03^a^	Additive

Each value is the mean of three replicates ± standard deviation (SD). The values for the individual standards with different letters in the same column and assay are significantly different (*p* < 0.05). The values for interactions between standards with different letters in the same file and assay are significantly different (*p* < 0.05). All units: *μ*mol TE/mL. Different superscript letters in the same column represent significant differences (*p* < 0.05) between individual hydroxycinnamic acids for TEAC or DPPH assay. Different superscript letters in the same row represent significant differences (*p* < 0.05) between real and theoretical values for each assay and each combination of hydroxycinnamic acids.
